# Quantitative MRI Dixon signal drop and fat fraction for differentiating bone marrow lesions: a two-center prospective analysis

**DOI:** 10.1186/s41747-025-00615-9

**Published:** 2025-09-10

**Authors:** Maha Ibrahim Metwally, Yassir Edrees Almalki, Marwa Fathy Khalil, Ahmed Mohamed Alsowey, Hazem Ibrahim Aly Tantawy, Mohamed Gaber Hamed, Shimaa Abdelmoneem, Sharifa Khalid Alduraibi, Ziyad A. Almushayti, Shaker Hassan S. Alshehri, Ahmed M. Abdelkhalik Basha, Mohammad Abd Alkhalik Basha

**Affiliations:** 1 Department of Radio-diagnosis, Faculty of Human Medicine, Zagazig University, Zagazig, Egypt; 2 Division of Radiology, Department of Internal Medicine, Medical College, Najran University, Najran, Kingdom of Saudi Arabia; 3 Medical Imaging Department, McMaster University, Faculty of Health Sciences, Hamilton, Ontario Canada; 4 Clinical Hematology Unit, Internal Medicine Department, Faculty of Human Medicine, Zagazig University, Zagazig, Egypt; 5 Department of Radiology, College of Medicine, Qassim University, Buraidah, Kingdom of Saudi Arabia; 6 Department of Orthopedic Surgery, College of Medicine, King Khalid University, Abha, Kingdom of Saudi Arabia; 7 Faculty of General Medicine, Saint Petersburg State University, Cairo Branch, Cairo, Egypt

**Keywords:** Bone marrow, Bone neoplasms, Biomarkers, Magnetic resonance imaging, Sensitivity and specificity

## Abstract

**Background:**

Bone marrow (BM) lesion differentiation remains challenging, and quantitative magnetic resonance imaging (MRI) may enhance accuracy over conventional methods. We evaluated the diagnostic value and inter-reader reliability of Dixon-based signal drop (%drop) and fat fraction percentage (%fat) as adjuncts to existing protocols.

**Materials and methods:**

In this prospective two-center study, 172 patients with BM signal abnormalities underwent standardized 1.5-T MRI protocols, including Dixon sequences. Two musculoskeletal radiologists independently evaluated images and performed quantitative measurements of %drop and %fat. Final diagnoses were established through histopathology (*n* = 96) or imaging follow-up (*n* = 76). Diagnostic value was assessed using area under the receiver operating characteristic curve (AUROC), inter-reader reliability using Cohen’s κ coefficient.

**Results:**

The consensus optimal cutoff was for %drop ≤ 19.8%, yielding 87.2% accuracy, 95.3% sensitivity, and 73.8% specificity, and that for %fat was ≤ 18.3%, achieving 86.6% accuracy, 96.3% sensitivity, and 70.8% specificity. Both metrics showed high diagnostic performance (AUROC 0.824–0.863) and excellent inter-reader reliability (κ > 0.93, *p* < 0.001). Multivariate analysis identified %drop ≤ 19.8% and %fat ≤ 18.3% as the strongest independent predictors of malignancy, with odds ratio (OR) being 9.38 and 8.85, respectively (*p* < 0.001). Signal characteristics on Dixon sequences provided additional diagnostic value, with signal voids on fat-only images (OR 7.14) and high signals on water-only images (OR 5.46).

**Conclusion:**

Quantitative MRI Dixon imaging parameters demonstrated high diagnostic accuracy and excellent inter-reader reliability in differentiating benign and malignant BM lesions, supporting their implementation in clinical practice protocols as a reproducible adjunct to conventional MRI.

**Relevance statement:**

Quantitative Dixon MRI provides reproducible, noninvasive differentiation of bone marrow lesions with high diagnostic accuracy across anatomical sites, enhancing clinical decision-making with standardized thresholds while demonstrating excellent inter-center consistency.

**Key Points:**

- Quantitative Dixon MRI thresholds of %drop ≤ 19.8% and %fat ≤ 18.3% were established as reliable predictors of malignancy in bone marrow lesions.
- Dixon metrics demonstrated superior diagnostic accuracy (86.6–87.2%), compared to conventional T1-weighted sequences (79.2%).
- Excellent inter-reader reliability (κ = 0.895–0.943) supports the reproducibility of quantitative Dixon MRI in clinical practice.

**Graphical Abstract:**

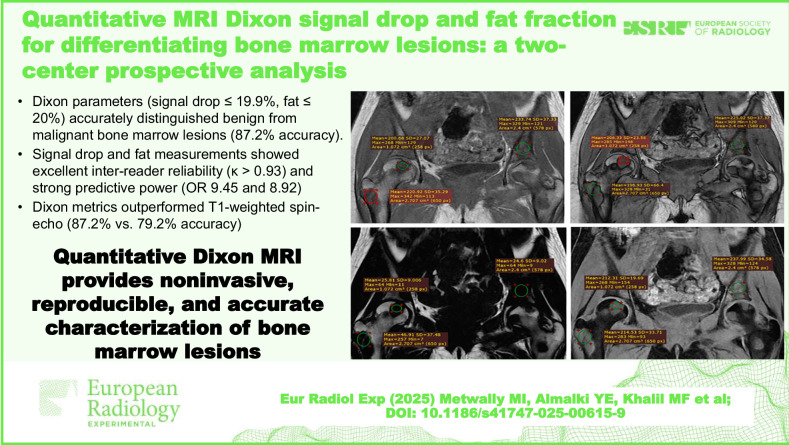

## Background

Abnormal bone marrow (BM) signals are frequently encountered in clinical practice and may indicate various pathological conditions. These include benign processes, such as BM edema, infection, stress reactions, and malignant entities, such as primary bone tumors and metastatic diseases [1]. Accurate differentiation between benign and malignant BM lesions is crucial for appropriate patient management and treatment planning [2]. Conventional imaging modalities, such as radiography and CT, have limited specificity for differentiating benign from malignant BM lesions because of overlapping imaging features [3]. Nuclear medicine studies, including bone scintigraphy and positron emission tomography-computed tomography (PET/CT), offer high sensitivity for detecting metabolically active lesions; however, their accessibility and associated high radiation exposure limit their routine use [4].

Magnetic resonance imaging (MRI) has emerged as a primary imaging tool for BM assessment due to its superior soft tissue contrast and multiplanar imaging capabilities, complementing histopathological diagnosis in clinical practice [5]. Conventional MRI sequences, including T1-weighted, T2-weighted, and short tau inversion-recovery images, provide valuable information regarding the BM composition and pathology [6]. While these conventional sequences remain essential for initial assessment, their qualitative nature often leads to diagnostic uncertainty in complex cases, highlighting the need for more quantitative approaches to BM evaluation. The Dixon technique, first described by William T. Dixon in 1984, is an advanced MRI method that allows quantitative fat-water separation using chemical shift imaging [7]. Since its introduction, this technique has undergone significant evolution, with technological advancements enabling robust quantification of fat fraction and improved tissue characterization, particularly for musculoskeletal applications, including BM imaging [8]. The technique’s ability to generate fat-only and water-only images, along with quantitative measurements of signal intensity (SI) reduction and fat content, makes the Dixon technique particularly valuable for BM assessment [8, 9].

Despite the well-documented advantages of Dixon imaging and the substantial body of literature supporting its implementation [10–22], standardized quantitative parameters for differentiating benign from malignant BM lesions have not been well established. Previous studies have relied primarily on qualitative assessments or limited quantitative analyses constrained by small sample sizes and single-institution experience [15, 19]. In recent years, targeted studies have explored its use in specific diseases such as vertebral metastases, pediatric marrow pathologies, leukemia differentiation, and fat quantification [23–28]. While studies like that by Bacher et al [29] have established the percentage of signal drop (%drop) and the percentage of fat fraction (%fat) cutoffs for specific contexts, such as vertebral fractures using 3-T scanners, broadly applicable, multicenter-validated parameters and their inter-reader reliability remain limited in clinical practice. Therefore, this prospective, multicenter study aimed to evaluate the diagnostic validity and inter-reader reliability of the %drop and the %fat as quantitative adjuncts to existing MRI protocols, enhancing the differentiation between benign and malignant BM lesions across diverse clinical settings.

## Materials and methods

This prospective study was approved by the Institutional Review Board (approval No: ZU—9485) and conducted following the principles of the Declaration of Helsinki. Written informed consent was obtained from all participants after a thorough explanation of the study protocol.

### Study design and population

**Fig. 1:**
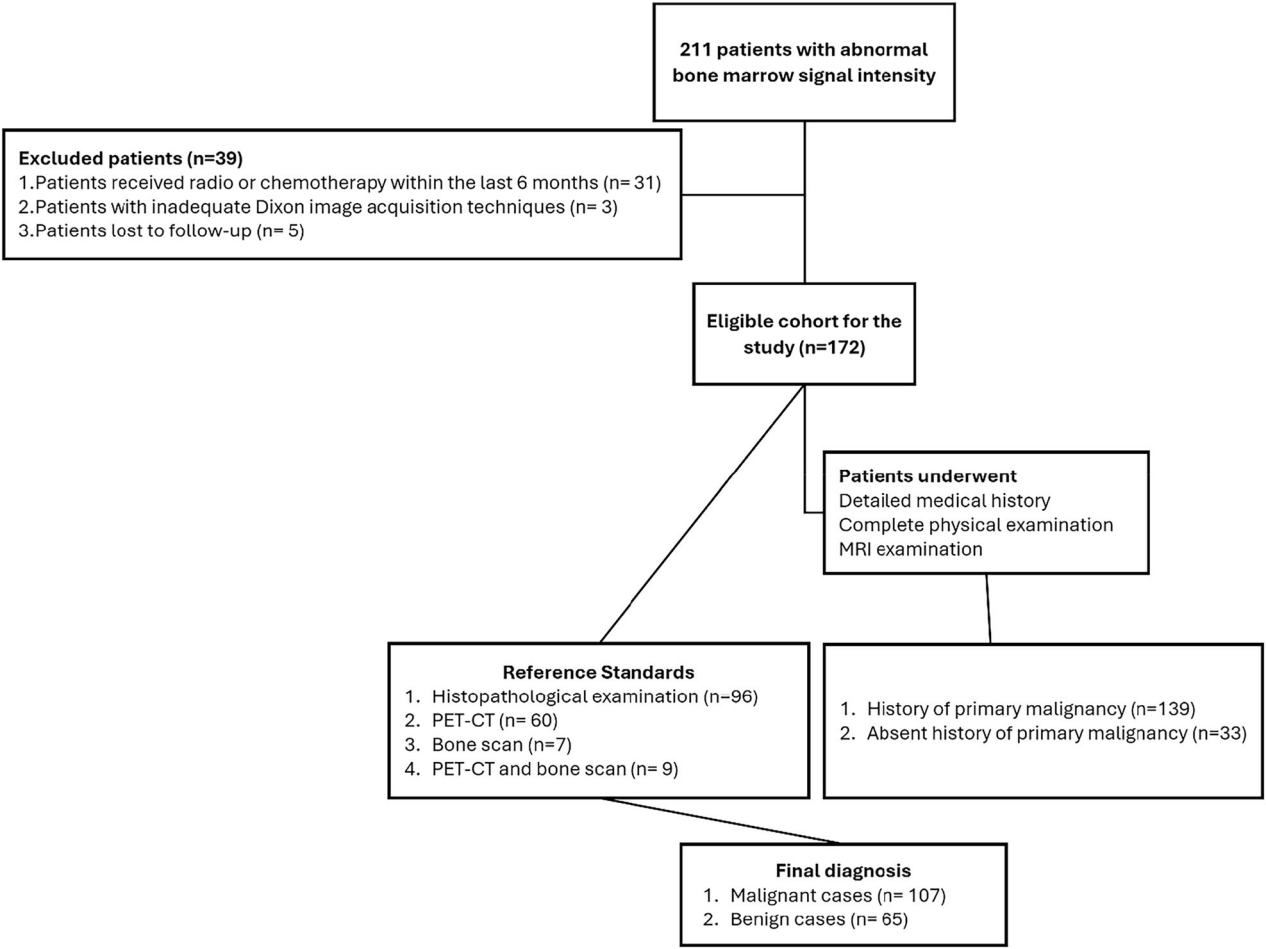
Study population flowchart showing patient selection process and exclusion criteria

This prospective multicenter study was conducted between September 2021 and February 2024. Initially, 211 participants with focal or diffuse BM signal abnormalities detected on conventional MRI of the axial skeleton or appendicular long bones were enrolled across two centers, designated center A and center B. Patients were excluded if they had received radiotherapy or chemotherapy within 6 months prior to imaging (*n* = 31), had technically inadequate Dixon image acquisition owing to motion artifacts or incomplete sequence acquisition (*n* = 3), or were lost to follow-up (*n* = 5). The final study cohort consisted of 172 subjects who underwent standardized MRI protocols, with 88 patients enrolled at center A and 84 at center B. The flowchart of the study is presented in Fig. 1.

### MRI protocol

All MRI examinations were performed using standardized protocols on two 1.5-Tesla systems: Philips Achieva class IIa (Philips Medical Systems) and GE Optima 450 GEM (GE Healthcare). Center A imaged 88 patients, using the Philips Achieva for 82 patients (93.2%) and the GE Optima for 6 patients (6.8%). Center B imaged 84 patients, using the GE Optima for 78 patients (92.9%) and the Philips Achieva for 6 patients (7.1%). Conventional sequences included unenhanced T1-weighted turbo spin-echo, T2-weighted turbo spin-echo, short tau inversion-recovery, diffusion-weighted imaging with *b*-values of 0 and 1,000 s/mm² (and their apparent diffusion coefficient maps). Contrast-enhanced imaging was performed using gadopentetate dimeglumine (0.1 mmol/kg at 2 mL/s), followed by T1-weighted sequences. Dixon imaging consisted of standardized unenhanced sagittal or coronal T1-weighted two-point Dixon sequences, generating in-phase, out-of-phase, water-only, and fat-only reconstructions. Specific acquisition parameters for Dixon sequences, including repetition time, echo time, flip angle, slice thickness, field of view, matrix, and scan time, were harmonized for both Philips and GE systems and are detailed in Table S1 in the supplementary materials, adapted from established musculoskeletal protocols [26, 30]. To minimize intersite and intervendor variability, standardized acquisition protocols were developed through a pre-study consensus between both centers’ radiology departments and implemented, with imaging performed within 48 h of clinical suspicion to ensure consistent timing. Monthly phantom calibration and quality assurance checks were conducted on both systems, using a standardized fat-water phantom to align signal intensity (SI) measurements as well as %drop and %fat calculations, with no significant differences in measurements between scanner models (*p* = 0.823 for %drop, *p* = 0.791 for %fat), ensuring consistency across Philips and GE platforms.

### Image analysis

Two musculoskeletal radiologists (M.F.K. and M.I.M., with 8 and 10 years of experience, respectively) conducted independent blinded evaluations. The SI was characterized in relation to skeletal muscle (low, intermediate, high, and heterogeneous) using T1-weighted, T2-weighted, short tau inversion-recovery, diffusion-weighted imaging sequences and apparent diffusion coefficient maps. Enhancement patterns were categorized as a non-enhancement, minimal, or moderate-to-intense enhancement. For Dixon sequence quantification, regions of interest (ROI; mean size 60 mm², range 50–100 mm²) were manually drawn by two radiologists to contour areas of maximal homogeneous signal abnormality on pre-contrast T1-weighted in-phase images, typically as circular or elliptical shapes, avoiding areas of necrosis, hemorrhage, calcification, and susceptibility artifacts. ROIs were drawn in one image and then copy-pasted in other images guided by pre-study training. For long bone lesions, the coronal or sagittal planes and the slice with the largest abnormal area were selected. SI measurements were performed for all four Dixon reconstructions.

The %drop and %fat were calculated using the following equations as adapted from Maeder et al [19]:

$$\% {{{\rm{drop}}}}= \,[({{{\rm{in}}}}{\mbox{-}}{{{\rm{phase}}}}\; {{{\rm{SI}}}} \, {\mbox{-}} \, {{{\rm{out}}}}{\mbox{-}}{{{\rm{of}}}}{\mbox{-}}{{{\rm{phase}}}}\; {{{\rm{SI}}}})/{{{\rm{in}}}} {\mbox{-}}{{{\rm{phase}}}}\; {{{\rm{SI}}}}]\times 100;$$

$$\% {{{\rm{fat}}}}= 	\,\left[\right.{{{\rm{fat}}}}{\mbox{-}}{{{\rm{only}}}}\; {{{\rm{image}}}}\; {{{\rm{SI}}}}/\left(\right.{{{\rm{fat}}}}{\mbox{-}}{{{\rm{only}}}}\; {{{\rm{image}}}}\; {{{\rm{SI}}}} \\ 	 {\mbox{+}} \, {{{\rm{water}}}}{\mbox{-}}{{{\rm{only}}}}\; {{{\rm{image}}}}\; {{{\rm{SI}}}}\left)\right.\left]\right.\times 100.$$

For patients with multiple lesions (*n* = 40) or diffuse lesions (*n* = 18), each radiologist independently placed three ROIs on representative areas of maximal homogeneous signal abnormality, guided by pre-study training, and the mean %drop and %fat values were calculated from these measurements for each patient. Following independent evaluations, both radiologists conducted consensus readings for all 172 cases, discussing features and measurements to standardize quantitative assessments and reduce subjectivity. Consensus findings determined diagnostic accuracy, with practical cutoff values derived from this joint assessment. Inter-reader reliability, calculated using Cohen’s κ on initial independent readings, reflected agreement on patient-level classifications based on mean %drop and %fat from three ROIs, not individual ROI coordinates.

### Reference standard

Final diagnoses were established using two approaches: (1) histopathological evaluation (*n* = 96) or (2) imaging follow-up (*n* = 76).

Histopathological assessment was obtained via image-guided core needle biopsy, *i.e*., minimum three cores per lesion, 14–18 gauge, targeting areas of maximum signal abnormality on Dixon MRI, performed within 7 days of imaging. Tissue samples were fixed in 10% neutral buffered formalin for 24 h, decalcified using ethylenediaminetetraacetic acid solution when necessary (for sclerotic lesions), embedded in paraffin, and sectioned at 4-μm thickness. Hematoxylin-eosin staining was performed as the primary assessment for cellularity, nuclear atypia, mitotic activity, and tumor infiltration patterns. Immunohistochemistry was performed in 75 cases (78.1%) for diagnostic confirmation or subtyping, with panels tailored to clinical suspicion: CD138, kappa/lambda light chains for suspected multiple myeloma; pan-cytokeratin (AE1/AE3), CK7/CK20, prostate-specific antigen for suspected carcinoma metastases; CD34, CD31 for vascular lesions; and CD68, CD163 for histiocytic/inflammatory conditions. Molecular testing was employed in 25 cases (26.0%) for challenging diagnoses, including fluorescence *in situ* hybridization for MYC, p53, and RB1 alterations in suspected malignancies, and polymerase chain reaction for microbial deoxyribonucleic acid in suspected infections. Two subspecialty-trained orthopedic oncology pathologists (> 10 years’ experience) independently evaluated all samples, with malignancy defined as either: (1) > 30% neoplastic cell infiltration, (2) unequivocal cytologic atypia with architectural distortion, (3) positive immunohistochemical markers with compatible morphology, or (4) specific molecular alterations indicative of malignancy. Benign lesions demonstrated non-tumorous marrow changes such as inflammation and edema, or benign neoplastic (*e.g*., hemangioma) features without meeting malignancy criteria. Inter-pathologist agreement was excellent (*κ* = 0.91, *p* < 0.001), with discrepant cases (*n* = 8) resolved through a consensus conference with a third musculoskeletal pathologist.

For cases without histopathological confirmation, imaging follow-up was performed using PET/CT (*n* = 60; standardized protocol with 6-h fasting, blood glucose < 150 mg/dL, ^18^F-fluorodeoxyglucose administration [5.5 MBq/kg], 60 min post-injection imaging), bone scintigraphy (*n* = 7; ^99m^Tc methylene diphosphonate [740 MBq] with planar imaging at 3 h post-injection and single-photon emission computed tomography/CT for focal abnormalities), or both (*n* = 9). Minimum follow-up duration was 12 months (range 12–24 months) with imaging performed at 3–6 month intervals. Malignancy was defined as progressive disease with increasing size/number of lesions or rising maximum standardized uptake value (> 25% increase), while benign lesions showed stability or regression. All cases underwent review by a multidisciplinary tumor board comprising radiologists, pathologists, and oncologists from both participating centers to ensure diagnostic consistency across histopathological and imaging-based diagnoses.

### Statistical analysis

Data analysis was performed using IBM SPSS 23.0 (IBM Corp.) and MedCalc 20.022 (MedCalc Software Ltd.). Normality was assessed using the Shapiro-Wilk test. Continuous variables are expressed as means, standard deviations, and ranges, while categorical variables are presented as frequencies and percentages. Comparative analyses included Fisher’s exact test, independent samples *t*-test, and Mann–Whitney *U* test, as appropriate. Diagnostic performance was evaluated using the area under the receiver operating characteristic curve (AUROC) analysis with optimal cutoff determination. Independent predictors were identified using multivariate logistic regression analysis, and the results were expressed as odds ratios (OR) with 95% confidence intervals. Inter-reader reliability was assessed using the Cohen’s κ coefficient, with values interpreted as poor (0.01–0.20), fair (0.21–0.40), moderate (0.41–0.60), good (0.61–0.80), and excellent (0.81–1.00). Statistical significance was defined as *p* < 0.05.

The overall sample size (*n* = 172) was determined a priori to achieve > 90% power to detect an AUROC ≥ 0.85 for %drop and %fat (*versus* null AUROC 0.70, α = 0.05), based on pilot data and prior studies [23], exceeding the minimum of 70 patients required for 80% power.

## Results

### Demographic and clinical characteristics

The final study cohort included 172 patients with focal and diffuse abnormal BM signals on the conventional MRI (Table 1). The patients had a mean age of 58.9 ± 17.3 years (range 8–93 years), with male predominance (72.1%). Back pain was the most common presenting symptom (62.2%), followed by pelvic pain (36.0%). Most patients (80.8%) had a history of primary malignancy, with prostate cancer being the most common malignancy (36.0%). Patients were distributed nearly evenly between center A (*n* = 88) and center B (*n* = 84), with no significant differences in demographic or clinical characteristics between sites (*p* = 0.894). The final diagnosis revealed malignant lesions in 107 patients (62.2%), with metastatic prostate cancer most frequent (36.0%). A subgroup analysis of benign lesions (37.8%) revealed benign bone tumors and non-tumorous changes such as spondylodiscitis, bone marrow edema, and myelofibrosis, with hematopoietic marrow reconversion most common (9.8%).

**Table 1:** Clinicopathologic characteristics of patients and bone marrow lesions

Characteristic	Value
Total number of patients	172
Age, (years), mean ± standard deviation (range)	58.9 ± 17.3 (8–93)
Sex	
Male	124 (72.1)
Female	48 (27.9)
Clinical presentation	
Back pain	107 (62.2)
Pelvic pain	62 (36.0)
Recurrent infection	14 (8.1)
Incidental finding	14 (8.1)
Primary malignancy	
Absent	33 (19.2)
Present	139 (80.8)
Breast cancer	5 (2.9)
Hepatocellular carcinoma	5 (2.9)
Multiple myeloma	12 (7.0)
Prostatic cancer	62 (36.0)
Rectal cancer	19 (11.0)
Colon cancer	5 (2.9)
Other malignancy	31 (18.0)
Reference standard	
Histopathology	96 (55.8)
PET/CT	60 (34.9)
Bone scan	7 (4.1)
PET/CT and bone scan	9 (5.2)
Final diagnosis	
Benign	65 (37.8)
Hematopoietic marrow reconversion	17 (9.8)
Lipid-poor hemangioma	10 (5.8)
Spondylodiscitis	8 (4.7)
Myelofibrosis	6 (3.5)
BM edema	14 (8.1)
Other benign	10 (5.8)
Malignant	107 (62.2)
Metastatic prostate cancer	62 (36.0)
Multiple myeloma	12 (7.0)
Metastatic rectal cancer	19 (11.0)
Metastatic breast cancer	5 (2.9)
Other metastases	9 (5.2)

Unless otherwise indicated, data are presented as number (percentage). Some patients presented with multiple findings

*PET/CT* positron emission tomography/computed tomography

### Lesion number and anatomical location

The cohort (*n* = 172) displayed diverse lesion patterns (Table S2), with single lesions most frequent (44.2%), followed by multiple lesions (23.3%), two lesions (18.0%), diffuse lesions (10.5%), and three lesions (4.1%). The spine was the primary site (52.3%), with stable involvement across types (50.0–57.1%). Long bones affected 50 patients (29.1%), with notable representation in single (28.9%), two (29.0%), and multiple (30.0%) patients. The pelvis, with 32 patients (18.6%), showed slightly higher proportions in diffuse (22.2%) and two-lesion (19.4%) patients.

### T1-weighted spin-echo and Dixon image analysis of BM lesions

Analysis of T1-weighted spin-echo and Dixon sequences revealed distinct SI patterns between benign and malignant BM lesions (Table 2). Malignant lesions predominantly showed low SI on T1-weighted spin-echo (88.8%), while benign lesions varied: low SI (36.9%), intermediate SI (30.8%), and high SI (32.3%) (*p* < 0.001). Malignant lesions predominantly showed a low SI on in-phase images (88.8%), whereas benign lesions demonstrated a more variable pattern, although still primarily a low SI (66.2%). On out-of-phase images, most malignant lesions (91.6%) exhibited intermediate SI, in contrast to benign lesions, which showed a mixed distribution of low (33.8%) and intermediate (40.0%) SI. Fat-only images demonstrated the most distinctive pattern, with all malignant lesions (100%) showing signal voids, whereas most benign lesions (70.8%) maintained an intralesional high SI. On water-only images, malignant lesions predominantly displayed a high SI (93.5%), with the majority (71.0%) showing moderately high or fluid-like signals. A subgroup analysis of benign lesions revealed a higher %drop (65.2%) and %fat (52.3%) in benign tumors *versus* lower values (41.8% and 32.1%) in non-tumorous changes, reflecting differences in fat content (*p* < 0.010).

**Table 2:** Dixon MRI analysis of bone marrow lesions

Variables	Benign lesions (*n* = 65)	Malignant lesions (*n* = 107)	*p*-value
SI on T1-weighted spin-echo			< 0.001
Low	24 (36.9)	95 (88.8)	
Intermediate	20 (30.8)	6 (5.6)	
High	21 (32.3)	6 (5.6)	
SI on in-phase image			0.040
Low	43 (66.2)	95 (88.8)	
Intermediate	10 (15.4)	9 (8.4)	
High	2 (3.1)	3 (2.8)	
Heterogeneous	10 (15.4)	0 (0)	
SI on out-of-phase image			< 0.001
Low	22 (33.8)	2 (1.9)	
Intermediate	26 (40.0)	98 (91.6)	
High	7 (10.8)	7 (6.5)	
Heterogeneous	10 (15.4)	0 (0)	
SI on fat-only image			< 0.001
Signal void	19 (29.2)	107 (100)	
Intralesional high SI	46 (70.8)	0 (0)	
SI on water-only image			< 0.001
Isointense	36 (55.4)	7 (6.5)	
High	29 (44.6)	100 (93.5)	
Mild high	19 (29.2)	24 (22.4)	
Moderate high/ fluid	10 (15.4)	76 (71.0)	
%drop, mean (range)	47.0 (1–81.6)	7.1 (-20.6 to 87.9)	< 0.001
%fat, mean (range)	46.2 (3.8–96.0)	9.5 (0.1–40.0)	< 0.001

*SI* Signal intensity, *%drop* Percentage of signal drop, *%fat* Percentage of fat fraction

### Diagnostic value of %drop and %fat in differentiating BM lesions

**Fig. 2:**
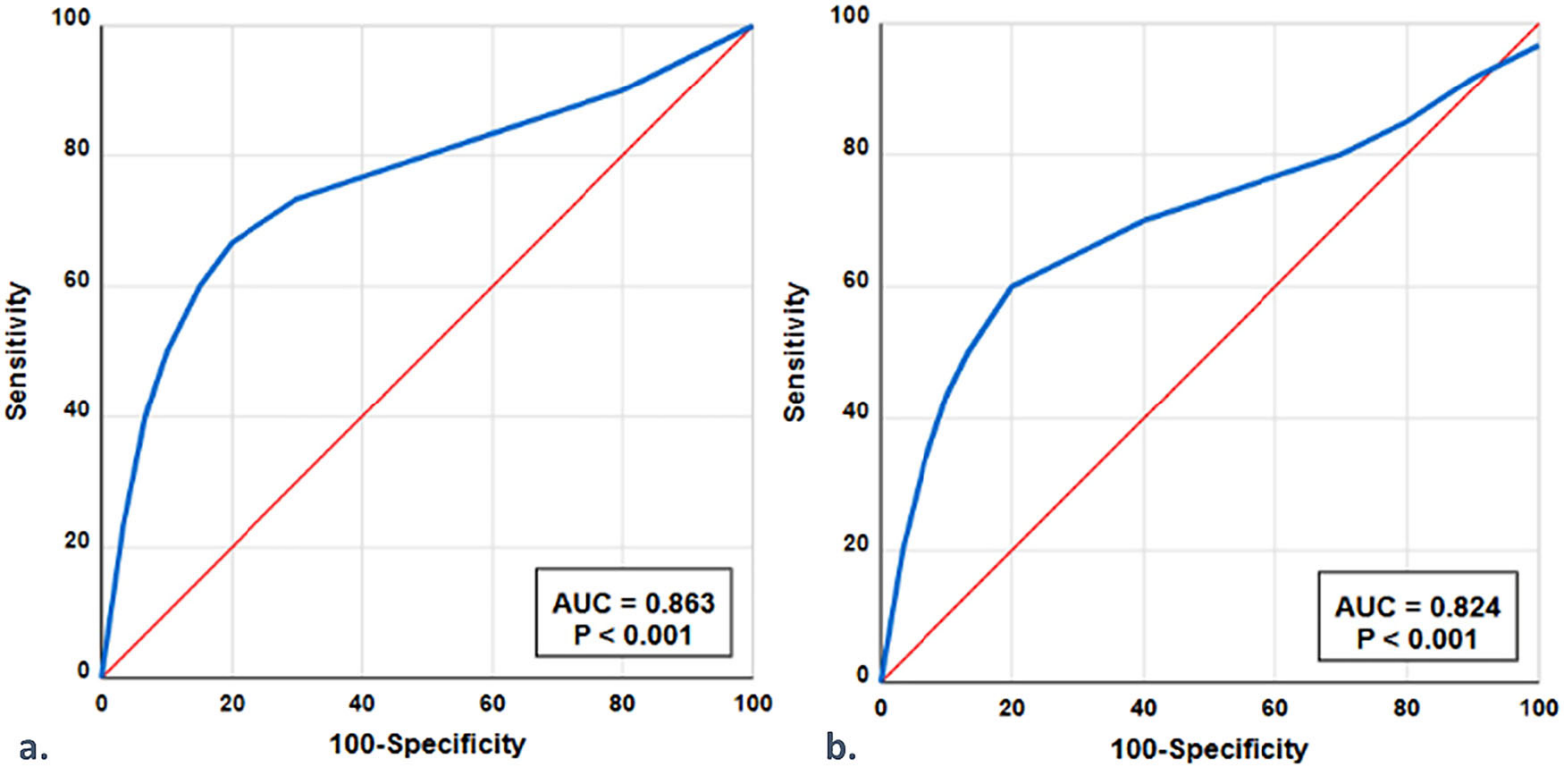
Diagnostic performance of Dixon MRI metrics for differentiating benign from malignant bone marrow lesions. **a** Percentage of signal drop with optimal cutoff value of ≤ 19.8%, sensitivity 95.3%, and AUROC 0.863; **b** Percentage of fat fraction with optimal cutoff value of ≤ 18.3%, sensitivity 96.3%, and AUROC 0.824. AUROC, Area under the receiver operating characteristic curve

AUROC analysis was performed to assess the diagnostic validity of %drop and %fat in differentiating BM lesions (Fig. 2, Table 3). The consensus optimal cutoff value for predicting malignancy was ≤ 19.8% for a %drop, yielding an accuracy of 87.2%, a sensitivity of 95.3%, and a specificity of 73.8%. The consensus optimal cutoff value for %fat was ≤ 18.3%, achieving an accuracy of 86.6%, a sensitivity of 96.3%, and a specificity of 70.8%. Both metrics demonstrated high diagnostic performance, with AUROC values of 0.863 for %drop and 0.824 for %fat.

**Table 3:** Diagnostic value of Dixon MRI metrics for differentiating bone marrow lesions

Parameters	Percentage of signal drop	Percentage of fat fraction
Cutoff value	≤ 19.8	≤ 18.3
AUROC	0.863 [0.753–0.928]	0.824 [0.715–0.903]
True positives (*n*)	102	103
False positives (*n*)	17	19
False negatives (*n*)	5	4
True negatives (*n*)	48	46
Accuracy (%)	87.2 (150/172) [77.5–94.1]	86.6 (149/172) [76.4–93.5]
Sensitivity (%)	95.3 (102/107) [84.9–99.5]	96.3 (103/107) [86.5–99.6]
Specificity (%)	73.8 (48/65) [53.7–88.9]	70.8 (46/65) [49.8–86.3]
Positive predictive value (%)	85.7 (102/119) [76.4–92.1]	84.4 (103/122) [75.1–90.7]
Negative predictive value (%)	90.6 (48/53) [71.7–97.5]	92.0 (46/50) [72.4–98.6]

Data in parentheses were used to calculate percentages. Data in brackets are 95% confidence intervals

*AUROC* Area under the receiver operating characteristic curve

However, the high sensitivity and moderate specificity (70.8–73.8%) of Dixon metrics prioritized detection of malignant lesions (positive predictive value 79.2% for %drop, 77.6% for %fat; negative predictive value 94.1% for %drop, 95.6% for %fat), with specificity limited by overlapping in benign conditions (*e.g*., spondylodiscitis *versus* malignancy). Using low SI as a malignancy criterion, the consensus reading of conventional T1-weighted spin-echo achieved 79.2% accuracy, 88.9% sensitivity, and 63.0% specificity. Compared with conventional T1-weighted spin-echo, Dixon-based metrics showed superior diagnostic performance across all parameters, suggesting potential added diagnostic value over T1-weighted spin-echo alone. A detailed comparison between T1-weighted spin-echo and Dixon parameters is presented in Table S3 in the supplementary materials. The consensus-derived cutoffs were consistent across both centers, with no significant inter-center variability in %drop (*p* = 0.823) or %fat (*p* = 0.791) measurements between the Philips Achieva and GE Optima scanners.

### Logistic regression analysis of Dixon MRI metrics in differentiating BM lesions

Multivariate logistic regression analysis identified six significant independent predictors of malignant BM lesions (Table 4). Quantitative metrics were the strongest predictors, with %drop ≤ 19.9% showing the highest OR (9.45, *p* < 0.001), followed by %fat ≤ 20% (OR 8.92, *p* < 0.001). Among the qualitative features, signal void on fat-only images demonstrated the highest predictive value (OR 7.14, *p* < 0.001), followed by high SI on water-only images (OR 5.46, *p* < 0.001) and intermediate SI on out-of-phase images (OR 5.12, *p* = 0.001). Low SI on in-phase images, although significant, had the lowest predictive value (OR 3.15, *p* = 0.005).

**Table 4:** Logistic regression analysis of Dixon MRI metrics in differentiating bone marrow lesions

Variable	Odds ratio	95% CI	*p*-value
Percentage of signal drop ≤ 19.8%	9.38	4.09–21.54	< 0.001
Percentage of fat fraction ≤ 18.3%	8.85	3.82–20.45	< 0.001
Signal void on fat-only image	7.14	3.09–16.52	< 0.001
High SI on water-only image	5.46	2.37–12.58	< 0.001
Intermediate SI on out-of-phase image	5.12	2.23–11.76	0.001
Low SI on in-phase image	3.15	1.42–6.98	0.005

CI Confidence interval, *SI* Signal intensity

### Inter-reader reliability

Inter-reader reliability analysis demonstrated excellent agreement (κ > 0.81, *p* < 0.001) across all the Dixon MRI metrics (Table 5). Fat-only image assessment showed the highest agreement (κ = 0.943), followed by quantitative measurements of %drop (κ = 0.935) and %fat (κ = 0.932). The SI assessment on in-phase (κ = 0.912), water-only (κ = 0.901), and out-of-phase images (κ = 0.895) also demonstrated excellent reliability. The overall diagnostic agreement between readers was excellent (κ = 0.921).

**Table 5:** Inter-reader reliability for Dixon MRI metrics

Measurement	κ-value (95% CI)
Percentage of signal drop	0.935 (0.845–1.000)
Percentage of fat fraction	0.932 (0.841–1.000)
In-phase image	0.912 (0.832–0.992)
Out-phase image	0.895 (0.815–0.975)
Fat-only image	0.943 (0.863–1.000)
Water-only image	0.901 (0.821–0.981)
Overall diagnostic agreement	0.921 (0.841–1.000)

**Fig. 3:**
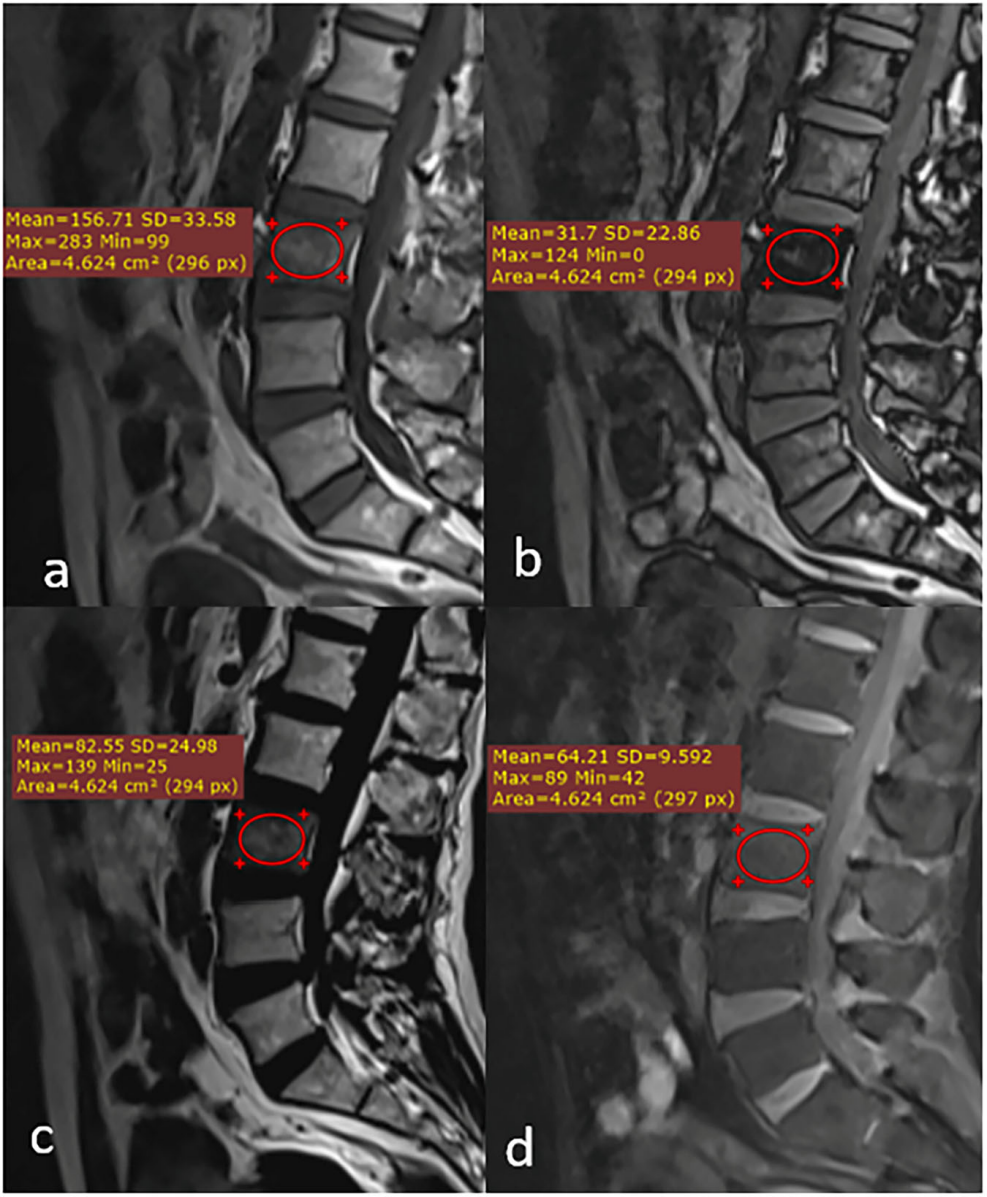
Dixon MRI sequences of a benign bone marrow lesion in a 72-year-old male. Sagittal images demonstrate: (**a**) heterogeneous signal intensity on the in-phase image (arrow); (**b**) marked signal drop on the out-of-phase image (arrow), indicating the presence of microscopic fat; (**c**) high signal intensity on the fat-only image confirming fat content (arrow); and (**d**) isointense signal on the water-only image (arrow). Quantitative measurements: signal drop (%drop) = 79.8% and fat fraction (%fat) = 56.2%. Final diagnosis: lipid-poor hemangioma, confirmed by negative bone scan and 6-month imaging stability

**Fig. 4:**
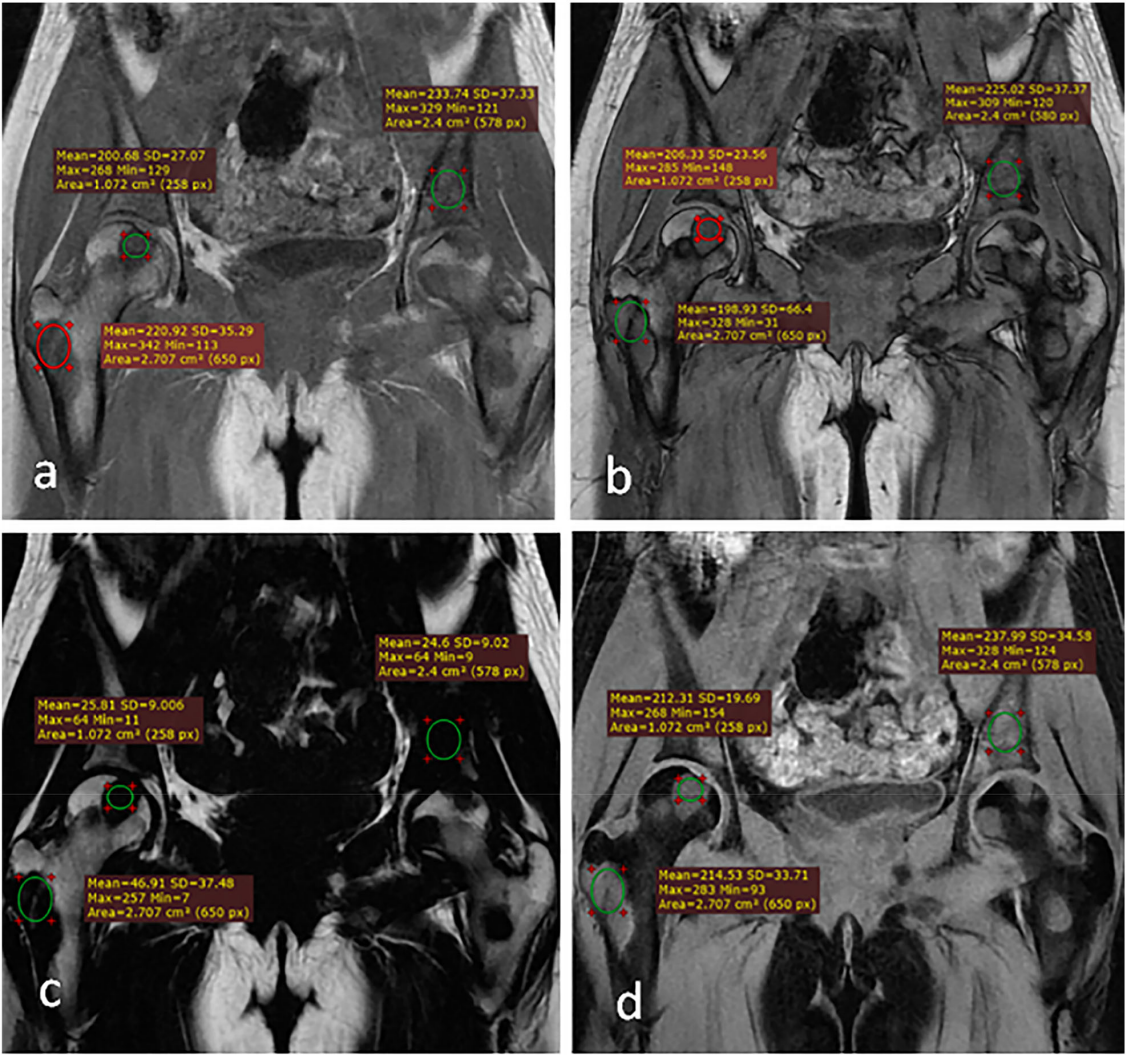
Dixon MRI sequences showing malignant bone marrow infiltration in a 15-year-old female with acute leukemia. Coronal images demonstrate: (**a**) multiple low signal intensity lesions on the in-phase image (arrows); (**b**) increased signal intensity of lesions on the out-of-phase image (arrows), indicating the absence of microscopic fat; (**c**) complete signal void on the fat-only image (arrows) suggesting replacement of normal marrow fat; and (**d**) high signal intensity on the water-only image (arrows) reflecting increased cellular content. Quantitative measurements: signal drop range = -2.6 to 9.9%; fat fraction range = 9.4 to 17.9%. Diagnosis confirmed by positron emission tomography/computed tomography

**Fig. 5:**
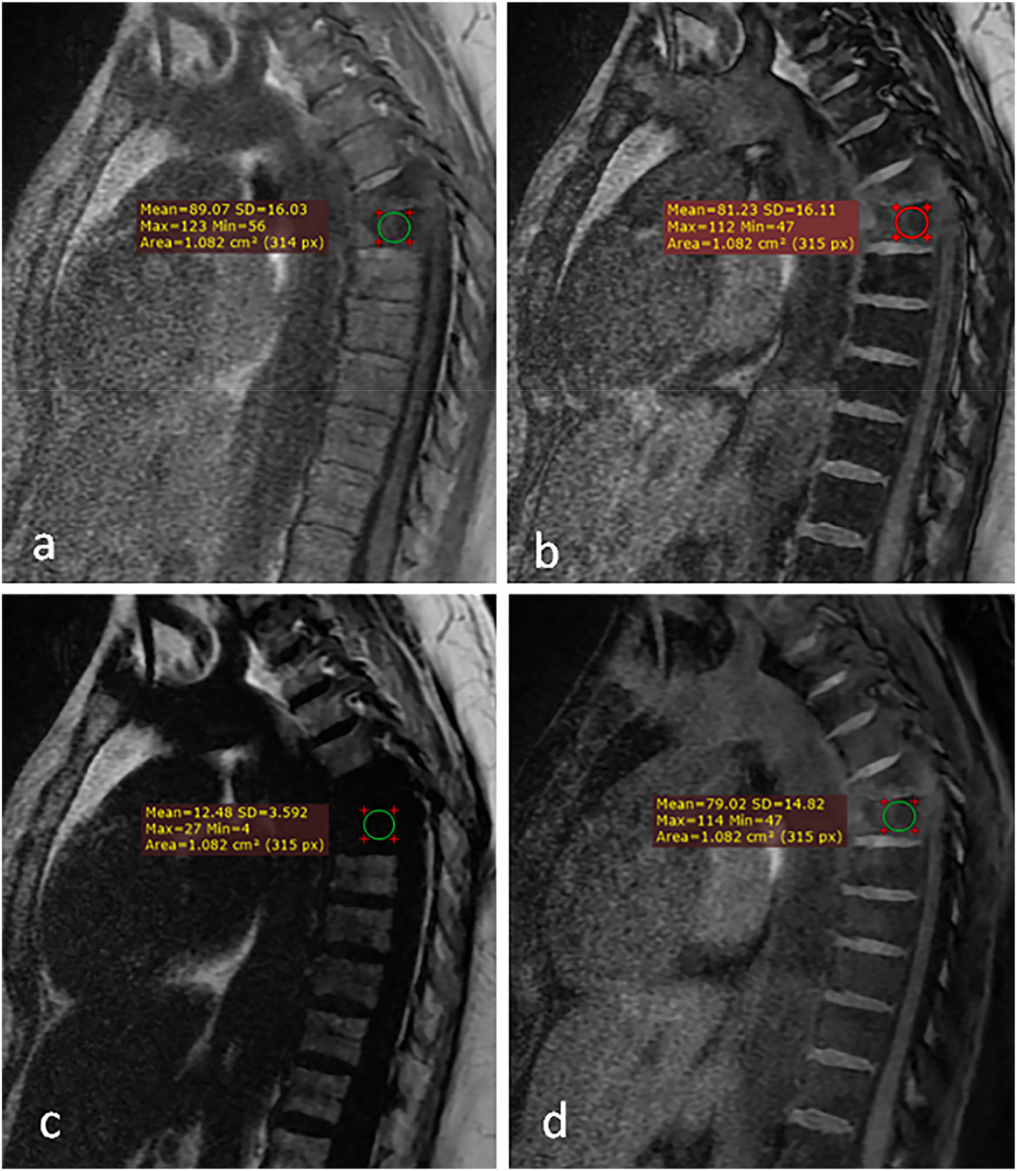
Dixon MRI sequences showing a false-positive case in a 43-year-old female with breast cancer history. Sagittal images demonstrate: (**a**) diffuse low signal intensity involving T5 and T6 vertebrae on the in-phase image (arrows); (**b**) increased signal intensity on the out-of-phase image (arrows) suggesting the absence of microscopic fat; (**c**) signal void on the fat-only image (arrows) mimicking complete marrow replacement, and (**d**) mildly increased signal intensity on the water-only image (arrows). Quantitative measurements: signal drop = 8.7%; fat fraction = 13.7%. Although imaging features suggested malignant infiltration, histopathology revealed grade II spondylodiscitis

Representative cases from our study are shown in Figs. 3, 4 and 5.

## Discussion

Differentiating between benign and malignant BM lesions remains a significant clinical challenge in musculoskeletal imaging. The most important outcome of our study was the establishment of precise MRI diagnostic thresholds using the Dixon MRI metrics for BM lesions. We found that a %drop of ≤ 19.8% and %fat of ≤ 18.3% effectively distinguished benign from malignant lesions with high accuracy. These findings validate previous research [11–15, 31, 32] and contribute substantially to clinical practice as the first large-scale, multicenter study to establish specific, quantifiable diagnostic thresholds for BM lesions. This study addressed the critical gap identified by Omoumi [13] by presenting standardized, evidence-based criteria for lesion characterization. Our defined thresholds align with the standardization efforts proposed by Guerini et al [16], offering reliable metrics that radiologists can use in routine diagnostic assessments.

The high sensitivity (95.3–96.3%) and moderate specificity (70.8–73.8%) observed in this study suggest that Dixon-based quantification effectively characterizes BM lesions. These results are consistent with those reported by Yoo et al [12], which were further extended to establish explicit diagnostic thresholds across a larger multicenter cohort in our study. This high sensitivity indicates that Dixon MRI is particularly valuable for diagnosing malignant lesions, especially in cases where early detection is crucial for patient outcomes. However, moderate specificity highlights the importance of using quantitative metrics alongside clinical judgment, especially when diagnosing benign lesions that may display imaging features similar to those of malignant lesions.

Our study found that malignant and benign lesions exhibited distinctly different signal patterns on Dixon sequences. On fat-only images, all malignant lesions (100%) showed signal voids, whereas most benign lesions (70.8%) maintained intralesional high SI. On water-only images, most malignant lesions (93.5%) displayed high SI, with 71.0% showing moderate high/fluid-like SI, while benign lesions showed a more variable pattern, with 55.4% appearing isointense. These patterns align with the fundamental principle of Dixon-based differentiation, which distinguishes between hematopoietic marrow (30–40% fat and 40–60% water) and fatty marrow (80% fat and 15% water) [19]. Quantitatively, benign lesions demonstrated a significantly higher mean %drop (47.0% *versus* 7.1%, *p* < 0.001) and %fat (46.2% *versus* 9.5%, *p* < 0.001) compared to malignant lesions. Malignant lesions that replace normal marrow and lack normal marrow fat do not produce a drop in signal on out-of-phase imaging, as evidenced by 91.6% showing intermediate SI on out-of-phase images, compared to only 40.0% of benign lesions [33–35]. These results reinforce the findings of previous studies and provide further diagnostic support for the use of Dixon MRI for evaluating BM lesions.

Quantitative analysis demonstrated statistically significant differences between benign and malignant lesions. Benign lesions had a higher %drop (47.0%) and %fat (46.2%) than malignant lesions (%drop: 7.1%; %fat: 9.5%). These differences align with the parametric ranges reported by Ogiwara et al [15] and confirm the reliability of Dixon MRI in distinguishing between these lesion types. Malignant lesions consistently showed a signal void on fat-only images (100%) and a high signal on water-only images (93.3%), which is consistent with the findings of Maeder et al [19] regarding BM metastases. This pattern recognition, combined with quantitative measurements, enhances diagnostic confidence and provides valuable and reproducible data for clinical practice. However, the wide %fat range in benign lesions (3.8–96.0%) reflects variability that complicates specificity without a normal BM reference.

Compared to T1-weighted spin-echo’s accuracy of 79.2%, Dixon metrics offered improved performance (87.2% for %drop, 86.6% for %fat), suggesting their potential as quantitative adjuncts to conventional assessment. These cutoffs provide standardized parameters with high reproducibility across readers and centers (κ > 0.93), though they prioritize malignancy detection (positive predictive value 77.6–79.2%) over normal BM exclusion.

Building on recent targeted studies [23–28], our work establishes reproducible cutoffs applicable across diverse clinical settings and anatomical sites, broadening the quantitative Dixon approach beyond vertebral-specific contexts. While our findings validate the diagnostic utility of Dixon parameters, it is important to note the differences between our established thresholds (%drop ≤ 19.8%, %fat ≤ 18.3%) and those reported by Bacher et al [29] for vertebral compression fractures (%drop ≤ 25%, %fat ≤ 20%). These discrepancies likely reflect methodological differences, including our use of 1.5-T scanners *versus* their 3-T platform, our T1-weighted Dixon protocol *versus* their T2-weighted approach, and our broader anatomical scope *versus* their vertebral-specific focus. Our study included a substantial spinal component (52.3%), allowing meaningful comparison with Bacher’s vertebral findings. However, our inclusion of long bones (29.1%) and pelvis (18.6%) represents an important extension of Dixon parameter validation beyond the axial skeleton. The slightly lower thresholds in our study may reflect these anatomical differences, as marrow composition varies across skeletal regions. A notable observation was the wide range of %drop values in malignant lesions, particularly the presence of negative values, which indicate paradoxical signal enhancement on out-of-phase sequences compared to in-phase sequences. These negative values correlate with the underlying matrix mineralization in some cases, as described by van Vucht et al [36]. However, this reversed ratio was also observed in other lytic lesions in our study and cannot be considered a specific characteristic of sclerotic lesions.

Analysis of misclassified cases revealed 17 false positives and 5 false negatives among our 172 patients. False positives predominantly occurred in benign marrow-replacing conditions—including myelofibrosis, spondylodiscitis, fibrous cortical defect, and non-ossifying fibroma—where tissue replacement mimicked malignant features [37]. Conversely, false negatives appeared in early-stage metastatic disease where insufficient malignant infiltration fell below detection thresholds [38, 39]. This pattern illustrates a critical diagnostic dilemma: the overlap in %drop and %fat values between benign marrow-replacing processes (*e.g*., spondylodiscitis with a %drop of 8.7%) and early-stage malignancies, reflecting limitations of derived parameters to capture subtle histopathological differences despite our prospective design’s controlled conditions. Similar challenges have been documented in pediatric lesions and skeletal metastases [24, 40], where fat fraction variability complicates differentiation. While Dixon metrics improve differentiation over T1-weighted spin-echo, their integration into diagnostic algorithms remains constrained by this variability, necessitating adjunctive imaging, particularly PET/CT [41], and clinical correlation rather than complete algorithmic reliance on these parameters. Recent multicenter studies [41, 42] suggest whole-body Dixon protocols may mitigate some variability, potentially informing future standardization efforts.

Multivariate analysis identified %drop ≤ 19.8% and %fat ≤ 18.3% as the most significant independent predictors of malignancy (OR 9.38 and 8.85, respectively, *p* < 0.001). Signal characteristics on fat-only and water-only images also yielded additional diagnostic values (OR 7.14 and 5.46, respectively), supporting the comprehensive utility of the Dixon technique as a reliable method for differentiating BM lesions, as described by Guerini et al [16]. These findings suggest that Dixon MRI metrics could be used not only to detect lesions but also to assess their malignant potential with high accuracy.

Our study’s excellent inter-reader reliability (κ = 0.895–0.943) further underscores the reproducibility of the Dixon measurements across different readers. This level of consistency is crucial for clinical practice, particularly as Dixon MRI techniques have become more widely implemented in routine diagnostic work. Our results align with the findings of Sasiponganan et al [10], who reported high reproducibility of quantitative signal alterations on Dixon sequences. This reinforces the potential of Dixon MRI as a robust and standardized tool for BM lesion evaluation.

Despite its strengths, this study has limitations. The overlap in %drop and %fat values between benign marrow-replacing processes and early malignancies remains a challenge, limiting specificity in ambiguous cases despite consensus cutoffs. Our reliance on derived parameters rather than direct fat quantification introduces potential variability from field inhomogeneity and partial volume effects. While outperforming T1-weighted spin-echo, we lack comparison to modalities like PET/CT. Additional limitations include potential intersite variability despite standardization efforts, vendor-specific algorithm differences between Philips and GE systems, and inconsistencies from dual reference standards. The absence of a healthy control group prevented establishing absolute normal thresholds, and our inter-reader reliability assessment with only two radiologists may overestimate reproducibility in clinical practice. Finally, our exclusive use of 1.5-T systems potentially limits generalizability to higher field strengths.

In conclusion, quantitative Dixon MRI metrics showed superior diagnostic performance compared to T1-weighted spin-echo and high reproducibility in differentiating BM lesions, indicating their potential as supportive clinical tools within existing MRI protocols rather than a comprehensive upgrade to diagnostic algorithms. The study highlights their practical utility across diverse settings, though limitations in derived parameters suggest cautious application. Future research should validate quantitative Dixon MRI across various MRI field strengths, pathological subtypes, and longitudinal studies, while enhancing protocol standardization and comparing it with other imaging methods to solidify its reliability for routine BM lesion evaluation.

## Supplementary information

**Additional file 1:** **Table S1** Parameters of Dixon image acquisition on Philips and GE scanners. **Table S2** Lesion number and anatomical location in the study cohort (*n* = 172). **Table S3** Comparative diagnostic performance of T1-weighted spin-echo *versus* Dixon parameters for bone marrow lesions.

## Data Availability

The datasets are available from the corresponding author upon reasonable request.

## Abbreviations

%drop: Percentage of signal drop
%fat: Percentage of fat fraction
AUROC: Area under the receiver operating characteristic curve
BM: Bone marrow
CT: Computed tomography
MRI: Magnetic resonance imaging
OR: Odds ratio
PET/CT: Positron emission tomography/computed tomography
ROI: Region of interest
SI: Signal intensity
