# Supplementary material for: Quantitative MRI Dixon signal drop and fat fraction for differentiating bone marrow lesions: a two-center prospective analysis
Source: Eur Radiol Exp. 2025 Sep 10;9:89. doi: 10.1186/s41747-025-00615-9 (PMC12423374; doi:10.1186/s41747-025-00615-9)
Supplement: Supplementary file 1 — Additional file 1: Table S1 Parameters of Dixon image acquisition on Philips and GE scanners. Table S2 Lesion number and anatomical location in the study cohort (n = 172). Table S3 Comparative diagnostic performance of T1-weighted spin-echo versus Dixon parameters for bone marrow lesions. [file 41747_2025_615_MOESM1_ESM.pdf]

Quantitative MRI Dixon signal drop and fat fraction for differentiating bone marrow lesions: a two-center prospective analysis

ELECTRONIC SUPPLEMENTARY MATERIAL

Table S1 Parameters of Dixon image acquisition on Philips and GE scanners

Parameters	Philips			GE		
	Cervical spine	Thoracic/lumbar spine	Pelvis	Cervical spine	Thoracic/lumbar spine	Pelvis
Repetition time (ms)	25	25	25	7.3	7.3	7.3
Echo time (ms)	4.6	4.6	4.6	4.8	4.8	4.8
Flip angle (degrees)	30	30	30	12	12	12
Number of excitations	1	1	1	1	1	1
Thickness (mm)	3	5	4	3	5	4
Interslice gap (mm)	0.6	1.2	1	0.6	1.2	1
Field of view (mm)	250 × 250	330 × 330	320 × 360	250 × 250	330 × 330	320 × 360
Matrix	226 × 256	226 × 256	192 × 256	226 × 256	226 × 256	192 × 256
Scan time (min:s)	1:43	1:50	2:58	1:55	2:05	2:50

Parameters were harmonized across both centers through a pre-study consensus to minimize inter-vendor variability, with monthly phantom calibrations using a standardized fat-water phantom to ensure consistency in signal intensity measurements, as detailed in the Methods section ($p = 0.823$ for %drop and $p = 0.791$ for %fat, Mann-Whitney *U* test)

Table S2 Lesion number and anatomical location in the study cohort (*n* = 172)

Lesion type	Total	Spine	Long bones	Pelvis
Single lesion	**76 (44.2)**	**40 (52.6)**	**22 (28.9)**	**14 (18.4)**
Two lesions	**31 (18.0)**	**16 (51.6)**	**9 (29.0)**	**6 (19.4)**
Three lesions	**7 (4.1)**	**4 (57.1)**	**2 (28.6)**	**1 (14.3)**
Over three lesions	**40 (23.3)**	**21 (52.5)**	**12 (30.0)**	**7 (17.5)**
Diffuse lesions	**18 (10.5)**	**9 (50.0)**	**5 (27.8)**	**4 (22.2)**
Total	**172 (100)**	**90 (52.3)**	**50 (29.1)**	**32 (18.6)**

Data are number of patients with the percentage in parentheses

Table S3 Comparative diagnostic performance of T1-weighted spin-echo *versus* Dixon parameters for bone marrow lesions

Parameter	Sensitivity (%)	Specificity (%)	Accuracy (%)
T1-weighted spin-echo (low signal intensity)	88.9 (95/107)	63.0 (41/65)	79.2 (136/172)
Dixon metrics			
Percentage of signal drop ≤ 19.8%	95.3 (102/107)	73.8 (48/65)	87.2 (150/172)
Percentage of fat fraction ≤ 18.3%	96.3 (103/107)	70.8 (46/65)	86.6 (149/172)
